# Treatment of malignant perivascular epithelioid cell tumor (PEComa) on the knee with an anterolateral thigh free flap: A case report

**DOI:** 10.1097/MD.0000000000034679

**Published:** 2023-08-11

**Authors:** Beom Jin Lim, Si-Gyun Roh, Jin Yong Shin, Nae-Ho Lee, Yoon Kyu Chung, Kyu Yun Jang

**Affiliations:** a Department of Plastic and Reconstructive Surgery, Medical School of Jeonbuk National University, Jeonju, Republic of Korea; b Research Institute of Clinical Medicine of Jeonbuk National University-Biomedical Research Institute of Jeonbuk National University Hospital, Jeonju, Republic of Korea; c Department of Pathology, Medical School of Jeonbuk National University, Jeonju, Republic of Korea.

**Keywords:** PEComa, perivascular epithelioid cell tumor, soft tissue tumor

## Abstract

The World Health Organization defines a perivascular epithelioid cell tumor (PEComa) as a mesenchymal neoplasia composed of perivascular epithelioid cells with characteristic morphological and immunohistochemical features. Although PEComas have the potential to behave in a malignant fashion, malignant PEComas are extremely rare.

**Patient concerns:**

An 83-year-old man visited our clinic presented with palpable, painless, and movable mass in the right knee area.

**Diagnoses:**

Malignant PEComa was diagnosed by incisional biopsy. No metastases was confirmed by radiologic imaging including PET/CT, magnetic resonance imaging, high resolution computed tomography.

**Interventions:**

We performed wide excision of the mass and used an anterolateral thigh free flap to reconstruct the defect on the right knee.

**Outcomes:**

The permanent histopathology showed malignant PEComa was totally resected. The flap which was performed to cover the defect was survived and the patient discharge without any complications.

**Lessons:**

PEComa can metastasize to various anatomical regions. Although there is no established standardized treatment, radical resection is still considered the cornerstone of treatment. Rapid and appropriate defect coverage is important to improve the patient’s prognosis.

## 1. Introduction

A perivascular epithelioid cell tumor (PEComa) is defined as a mesenchymal neoplasia composed of perivascular epithelioid cells with characteristic morphological and immunohistochemical features in the fourth edition of the World Health Organization’s *Classification of Tumors of Soft Tissue and Bone.*^[1]^

PEComa is a rare disease, with approximately 100 cases reported worldwide.^[2]^ PEComa occurs in various anatomical regions, including the colon, pancreas, retroperitoneum, heart, adrenal gland, breast, eye, biliary tract, bone, urinary bladder, skull base, liver, uterus, cervix, skin, nasopharynx, upper airway, and soft tissue. Cases occurring in soft tissue are even rarer than those in other areas.^[3]^

Herein, we report a rare case of an 83-year-old male patient who presented with malignant PEComa on the right knee. We performed wide excision of the mass and used an anterolateral thigh free flap to reconstruct the defect on the right knee. This study received approval from the Jeonbuk National University Hospital’s institutional review board. The study was conducted in accordance with the principles of the Declaration of Helsinki, and the patient, being elderly and considered as vulnerable population, written informed consent was received from the patient’s legal guardian prior to inclusion in the study.

## 2. Case report

**Figure 1.**
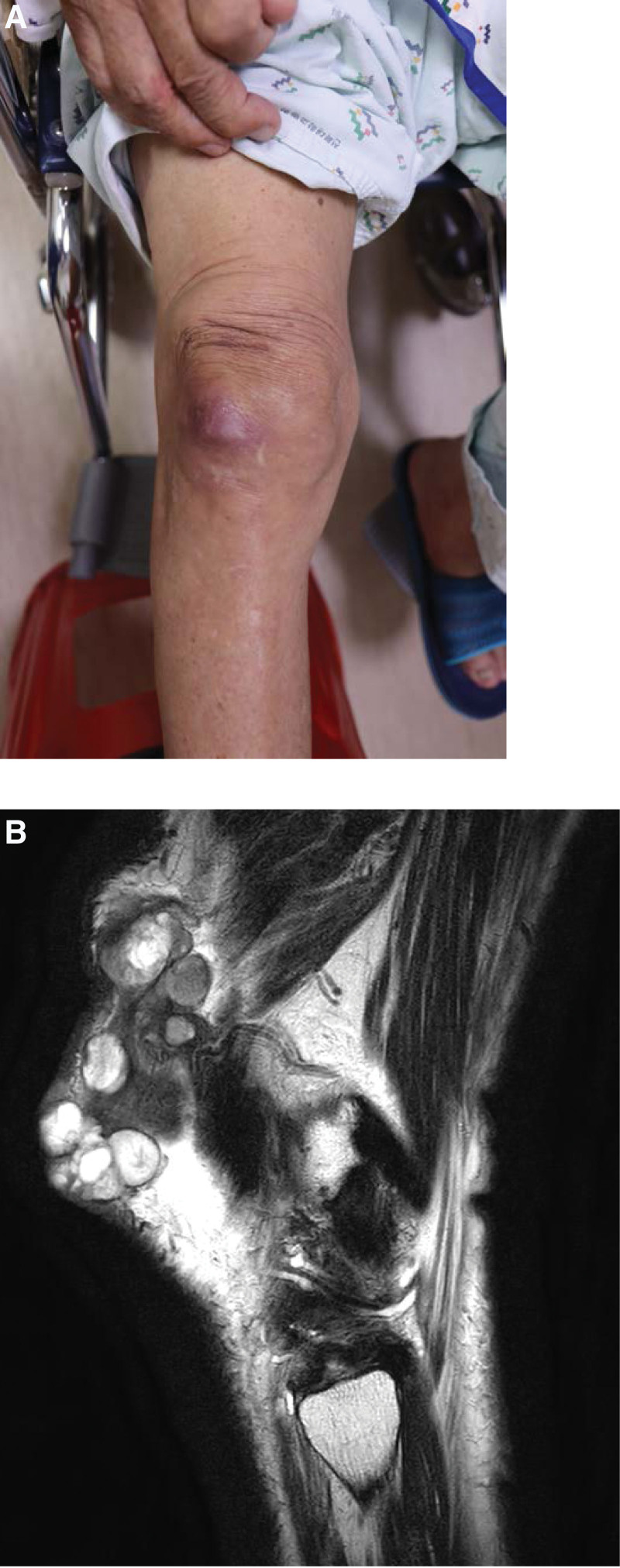
(A) About 12 × 10 cm sized mass was palpated on subcutaneous layer of right knee. (B) MRI view. MRI = magnetic resonance imaging.

An 83-year-old man visited the outpatient department with a palpable, painless, and movable mass in the right knee area. The patient had a history of gastric cancer that was completely cured after surgery. A biopsy was performed for evaluation, and PEComa was diagnosed. Subsequently, a bone scan, positron emission tomography-computed tomography, magnetic resonance imaging (MRI), and high-resolution computed tomography were performed to evaluate the lesion and determine whether metastasis had occurred. On MRI, multiple subcutaneous soft tissue masses with high density and enhancement were observed in the right prepatellar region, but no metastasis was observed (Fig. 1).

Wide resection was performed with a safety margin of 3 cm, and the lateral third of the patella was resected. After resection, there was a large skin and soft tissue defect measuring about 20 × 10 cm, and an anterolateral thigh free flap was planned to cover the defect. An elliptical flap was designed for the contralateral thigh, and a myocutaneous perforator originating from the descending branch of the lateral circumflex femoral artery was found and dissected using Doppler ultrasound.

**Figure 2.**
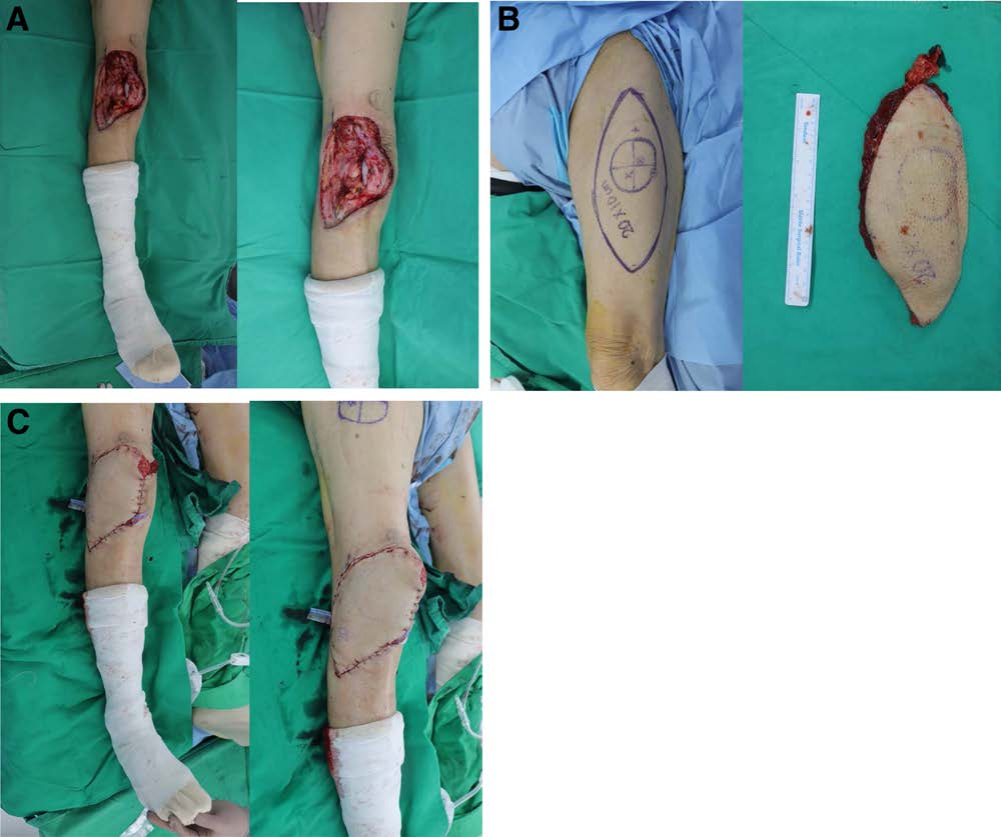
Surgical procedure of defect coverage with ALT free flap. (A) About 20 × 10 cm sized skin and soft tissue defect was observed. (B) Anterolateral thigh flap was designed on left thigh. (C) Defect on right knee was covered with ALT free flap.

The elevated flap was inserted into the defect site, and end-to-end anastomosis was performed between the descending branch of the lateral femoral circumflex artery and the medial genicular artery. An end-to-end anastomosis was performed between the vena comitans and the medial genicular vein (Fig. 2).

**Figure 3.**
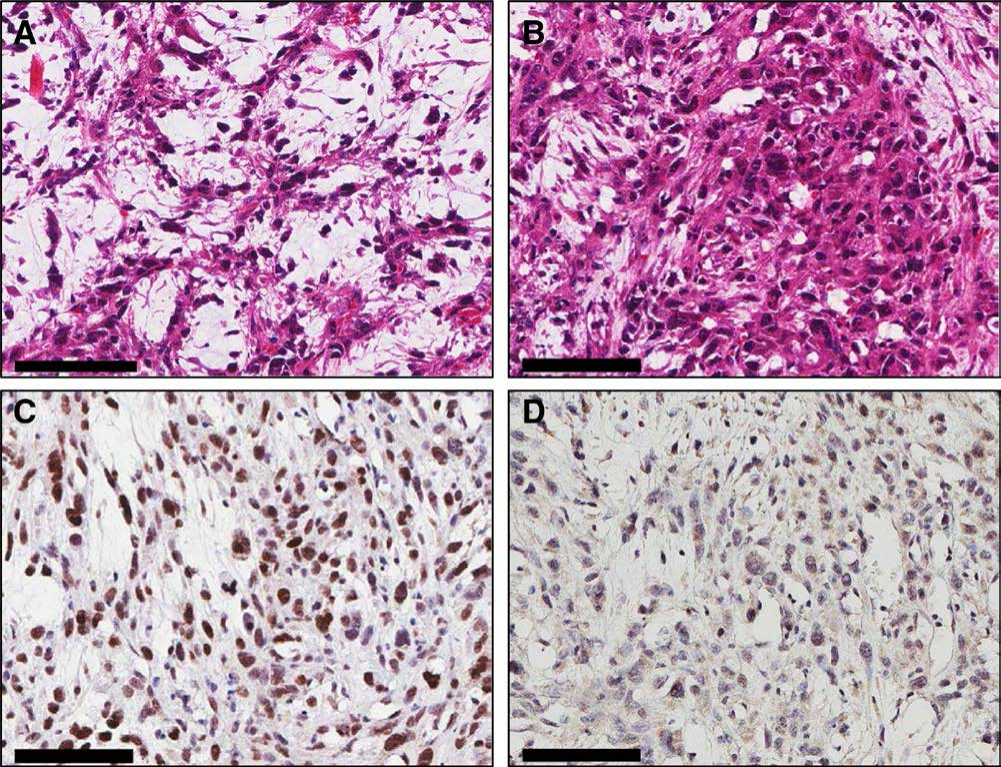
Histologic findings of malignant perivascular epithelioid cell tumor. (A) The tumor is composed of epithelioid cells, with clear cytoplasm, that form nests. (B) The tumor cells show significant cytologic atypia. (C and D) The tumor cells are strongly positive for TFE3 (C) and weakly positive for HMB45 (D). Scale bars: 100 μm. TFE3 = Transcription Factor Binding to IGHM (immunoglobulin heavy constant Mu) Enhancer.

**Figure 4.**
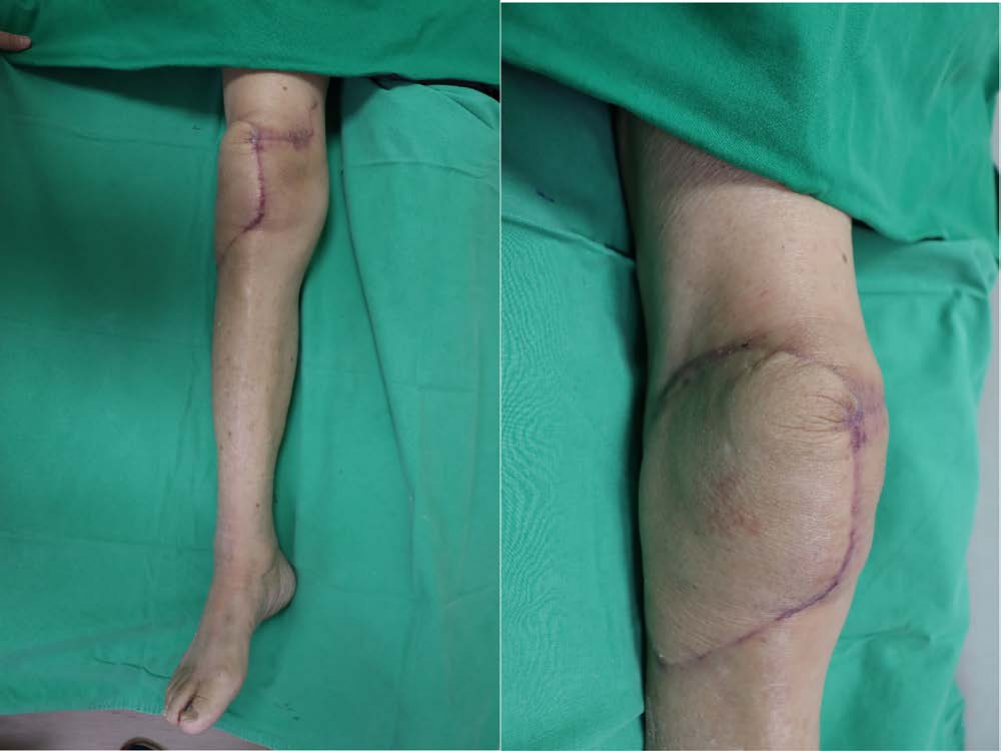
In follow-up period, there were no complications or metastases.

The permanent histopathology showed malignant PEComa which is composed of epithelioid cells, with clear cytoplasm and significant cytologic atypia. Also, the tumor cells showed strongly positive for Transcription Factor Binding to IGHM (immunoglobulin heavy constant Mu) Enhancer (TFE3) and weakly positive for HMB45 (Fig. 3). Appropriate post-free flap management was performed, including flap monitoring, and proper flap perfusion was maintained. Two weeks after surgery, the flap was confirmed to have survived, and the patient was discharged without any complications in the operated area. Since then, chemotherapy that could cause cytotoxicity was not administered in consideration of the patient’s history of previous gastric cancer and old age; however, focal radiation therapy was performed 10 times. Outpatient follow-up was continued and evaluations were periodically performed for recurrence and metastasis. The flap was well maintained, with no recurrence or flap site abnormalities (Fig. 4).

## 3. Discussion

PEComas are a rare tumors defined by the World Health Organization as a mesenchymal neoplasia composed of perivascular epithelioid cells with characteristic morphological and immunohistochemical features.^[2]^ PEComa occurs in various anatomical regions, including the colon, pancreas, retroperitoneum, heart, adrenal gland, breast, eye, biliary tract, bone, urinary bladder, skull base, liver, uterus, cervix, skin, nasopharynx, upper airway, and soft tissue.^[3]^ PEComa is mainly diagnosed between the ages of 38.9 and 56 years, but cases in children have also been reported, and it is known to occur more frequently in women (54–86.9%) than in men. The PEComa family includes angiomyolipoma, clear-cell sugar tumors, lymphangiomyomatosis, and clear cell myomelanocytic tumors, which are characterized by immunoreactivity with melanocytic and muscle markers.^[4]^

PEComas may be discovered by examining painful areas or palpable masses, but about 20% of cases are found incidentally on radiological imaging such as computed tomography or MRI taken for the purpose of diagnosing other diseases. Since PEComa can occur in various anatomical regions, it is important to perform a complete diagnostic evaluation, and a biopsy is essential for the diagnosis.^[2]^ The pathological findings are characterized by perivascular proliferation of epithelioid and fusiform cells with light eosinophilic cytoplasm with granularities and visible nucleoli. On immunohistochemical staining, about 92% to 100% of melanocytic markers are positive for HMB-45, 23% to 88% are positive for Melan A/Mart1, 50% to 92% are positive for the micropthalmia transcription factor, and 8% to 33% show positive findings for S100. As for the smooth muscle markers, about 36% to 100% are positive for desmin, 59% to 93% are positive for smooth muscle actin, and 75% to 92% are positive for caldesmon. In addition, regardless of the rearrangement of the *TFE3* gene, 29% to 38% of PEComas can show positive findings for the TFE3 transcription factor. PEComas can metastasize to adjacent lymph nodes, bones, and organs, and the most common organ for metastasis is the lung. Since metastasis may occur up to 10 years after surgical resection, regular follow-up is necessary.^[5–7]^

As PEComa is a rare disease, there is no established standardized treatment, but the main treatment is currently radical resection. Radical resection is recommended for metastatic tumors, but a poor prognosis is predicted. According to Machado study, palliative chemotherapy, radiation therapy, and immunotherapy can be considered in patients with metastasis. However, data are limited and this remains an area where no consensus exists, requiring more evidence.^[8]^ According to Bajaj et al, the activation of mammalian target of rapamycin signaling as a result of mutations or deletions of tuberous sclerosis genes (*TSC1* or *TSC2*) is a key molecular driver of PEComa. Their study introduced a case with an improved clinical response in a patient with metastatic malignant PEComa who received concurrent administration of a mammalian target of rapamycin inhibitor and radiation therapy. Therefore, those researchers pointed out that an appropriate treatment plan should be established after weighing the benefits and risks of cytotoxic chemotherapy in consideration of the patient’s age and underlying disease.^[9–11]^

## 4. Conclusion

PEComa is a rare disease and can metastasize to various anatomical regions. Although further research is required, radical resection is still considered the cornerstone of treatment, and there is no established treatment method due to the low prevalence of this condition. Choosing an appropriate coverage method for a large defect after radical resection can be challenging for surgeons, and free flap surgery can be a good option if the patient’s condition permits. Stabilization of the surgical site can allow rapid initiation of postoperative chemotherapy, radiation therapy, and immunotherapy; thus, rapid and appropriate defect coverage is important since it can affect the patient’s prognosis.

## Author contributions

**Conceptualization:** Nae-Ho Lee.

**Data curation:** Beom Jin Lim.

**Investigation:** Beom Jin Lim, Kyu Yoon Jang.

**Methodology:** Kyu Yoon Jang.

**Resources:** Beom Jin Lim.

**Supervision:** Si-Gyun Roh.

**Writing – original draft:** Beom Jin Lim, Si-Gyun Roh.

**Writing – review & editing:** Si-Gyun Roh, Jin Yong Shin, Nae-Ho Lee, Yoon Kyu Chung.
